# Effect of prebiotic supplementation with stabilized rice bran in milk of pre-weaned organic Holstein calves

**DOI:** 10.1186/s12917-019-1802-3

**Published:** 2019-02-07

**Authors:** Ana Velasquez-Munoz, Diego Manriquez, Sushil Paudyal, Hyungchul Han, Robert Callan, Elizabeth P. Ryan, Pablo Pinedo

**Affiliations:** 1 Department of Animal Sciences, Colorado State University, Fort Collins, CO 80523 USA; 2 Department of Clinical Sciences, College of Veterinary Medicine and Biomedical Sciences, Colorado State University, Fort Collins, CO 80521 USA; 3 Department of Environmental and Radiological Health Sciences, College of Veterinary Medicine and Biomedical Sciences, Colorado State University, Fort Collins, CO 80523 USA; 4 Animal and Veterinary Science Department, California Polytechnic University Pomona, Pomona, CA 91768 USA

**Keywords:** Neonatal diarrhea, Stabilized Rice bran, Prebiotic, Dairy Calves

## Abstract

**Background:**

The first month of life possess significant challenges for dairy calves due to high susceptibility to digestive diseases. The objective of this study was to evaluate the effect of prebiotic supplementation with stabilized rice bran (SRB) in milk on health, immunity, and performance of pre-weaned organic dairy calves. Holstein heifer calves (*n* = 90) were enrolled at 6 ± 1 days old and monitored for 28 days, from July to August 2017. Calves were randomly assigned to a control (CTR; *n* = 45) or a treatment group (SRB; *n* = 45). The CTR group received milk alone and the SRB group received 120 g of SRB per day in milk to achieve a 10% *w*/w dose of the total calories. Daily health evaluations were conducted to score health status and disease severity (healthy, slightly affected, moderately or severely sick) of calves, through integrated assessment of diarrhea, dehydration, attitude, and milk intake. Body weights and fecal IgA quantification were completed on the first and last day of the study.

**Results:**

Overall, weight gain and fecal IgA concentrations were not affected by the dietary addition of SRB. The total number of calf-days classified as healthy or sick were not different between treatment groups. Similarly, the number of calf-days categorized as slightly affected, moderately sick, or severely sick did not differ between treatment groups. Time to event analyses indicated a tendency for a treatment effect in the time to the first moderate case of diarrhea (*P* = 0.08), as well as in the time to recovery from diarrhea (*P* = 0.052), favoring control calves.

**Conclusions:**

These results indicated that the dietary addition of SRB in milk did not have an effect in health, immunity or performance of pre-weaned dairy calves.

## Background

Rearing healthy calves that maintain adequate growth rates is essential for the success of dairy operations. However, during the first month of life, calves face multiple stressors while the immune system is still developing, resulting in a high susceptibility to digestive diseases [1].

During the first weeks of life of dairy calves, diarrhea is the most prevalent health disorder, as well as the main cause of death. A recent report in the US indicated that 56.4% of calf mortality was a consequence of diarrhea and animals less than 4 weeks old were the most affected [2]. In 2013, 21% of pre-weaned calves presented diarrhea and 16% of all pre-weaned calves were treated with antimicrobials [2]. Rehydration and antibiotic therapy are common treatments for calves with neonatal diarrhea. However, due to consumer concerns, regulations for the use of antibiotics in food animals are becoming more restrictive. Consequently, research focused on alternatives to the use of antimicrobials, including strategies to prevent disease is required.

Prebiotics are defined as non-digestible feed ingredients that stabilize the intestinal microbiota, stimulating the growth of beneficial bacteria and inhibiting the colonization by pathogens [3, 4]. Prebiotic-probiotic interactions have been shown to improve immune responses [3, 5], contrasting with the action of antibiotics that eliminate and restrict the growth of detrimental and beneficial microorganisms with no distinction.

The use of prebiotics has been studied in young ruminants as a prophylactic strategy to prevent disease and as an alternative to antibiotics and one of the most common products is mannanoligosaccharides (MOS), a derivative of the cell wall of the yeast *Saccharomyces cerevisiae.* However, the effects of prebiotics on performance, health, and immunity of calves has not been consistent. For example, the supplementation of MOS resulted in a reduction in almost 1 point on the severity of neonatal diarrhea in a 1 to 4 scale [6] and decreased the number of days with high diarrhea scores [7]. Contrary, other studies reported no differences in diarrhea cases after the supplementation prebiotics [8–10] in pre-weaned dairy calves. Furthermore, some studies indicated no differences in weight gain [8, 10, 11], while greater gains were reported by others [6, 12, 13] after a prebiotic supplementation in pre-weaned calves.

Heat stabilized rice bran (SRB) contains prebiotics that have been tested in mice, chickens, pigs, horses, dogs and humans. This is a natural product that has been heat stabilized to prevent rancidity. As other prebiotics used in calf health, SRB is a carbohydrate. However, SRB contains ɣ-Oryzanol (omega 6–9), antioxidants (tocopherols, tocotrienols, polyphenols, phytosterols), vitamin E and B, amino acids (tryptophan, histidine, methionine, cysteine, arginine) and micronutrients (magnesium, calcium, phosphorus, manganese), which may have the potential to enhance the host health, not only through a symbiotic effect with the probiotic bacteria in GI tract [16]. Previous research indicated that this product had positive effects reducing the presentation and duration of diarrhea from human rotavirus and human norovirus in pigs [14], increasing the production of local and systemic IgA and enhancing the immune system in mice and pigs [14–17]. The effect of SRB has not been previously studied in young ruminants and its potential as a supplement or additive in whole milk of pre-weaned dairy calves has not been explored.

We hypothesized that the addition of SRB in milk of pre-weaned calves would reduce the presentation and severity of neonatal diarrhea, improving the immune response and consequently the overall calf performance. Therefore, our specific objective was to determine the effect of SRB on average daily gain (ADG), fecal IgA concentration, presentation of diseases, time to recovery from disease, and animal removal.

## Results

Overall, 88 calves were included for the final analyses, as 2 calves in the SRB group did not consume the milk with added SRB.

### Baseline

All calves had baseline total serum protein (TSP) measurements above 5.5 g/dL, indicating no failure in passive immune transfer [18, 19]. However, 31 calves had TSP measurements above 7.5 g/dL and from these, 23 presented diarrhea at the time of enrolment. Consequently, 35% of the enrolled animals might have presented some degree of dehydration that could alter to some extent the values of TSP. No significant difference (*P* = 0.94) was found for the proportion of TSP above 7.5 g/dL between control (CTR) and SRB calves (OR = 1.03, 95% CI = 0.43–2.47). Additionally, at enrollment, 43 (49%) calves presented signs of slight disease (diarrhea), which may also explain the high TSP level and possible dehydration. No differences (*P* = 0.39) were found in the odds (95% CI) of diarrheal disease at enrollment (OR = 1.44 [0.62–3.34]) for CTR calves in comparison with the SRB group.

### Health status, disease presentation and recovery time

**Fig. 1:**
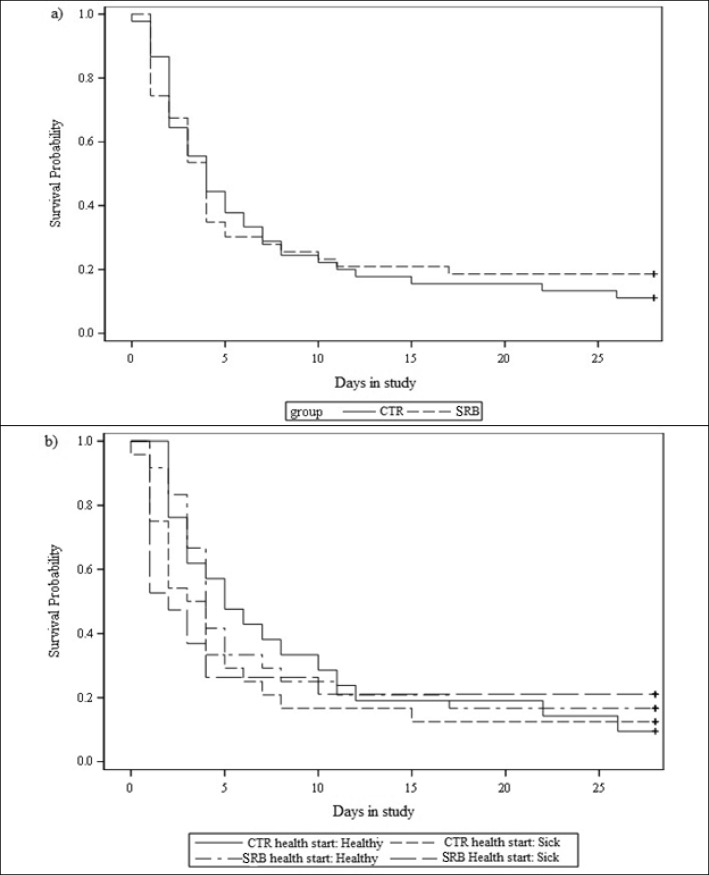
Time to occurrence of the first “moderate” diarrhea episode. **a** Treatment group (**b)** Health at enrollment and treatment group. Control (CTR; solid line) vs. Stabilized Rice Bran (SRB; dashed line) groups (*P* = 0.71). Comparison including health status at enrollment and treatment groups. Healthy CTR (solid line), healthy SRB (dashed and dotted line), sick CTR (dashed line), sick SRB (line and dashed line), (*P* = 0.08)

For the overall 28 day study, total calf-days classified as “healthy” and “sick” (“slight”, “moderate” or “severe”) for 88 calves were 1198 and 1230, respectively. These cumulative days were analyzed according to disease severity and dietary treatment group (Table 1). The repeated measures analyses for a binary response did not indicate a significant effect for treatment in the number of “healthy” or “sick” days for any of the disease categories (Table 2). No differences between treatment groups were found for the time to the first “moderate” disease event (*P* = 0.71). The survival curve demonstrated a pronounced slope (Fig. 1a) during the first 5 days of study, with about 70% of SRB calves and 60% of CTR calves presenting the first “moderate” disease status within this period. When the health status at enrollment was included as a covariate in the analysis, a tendency was determined in the survival function for the effect of health at day 0 (*P* = 0.08; Fig. 1b). Day 0 “healthy” CTR and SRB calves presented the first moderate subsequent diarrhea episode in 9 ± 2 days and 7 ± 1 days respectively. Contrary, d0 “sick” CTR and SRB presented the first moderate diarrhea episode in 5 ± 1 and 4 ± 1, respectively.

**Table 1:** Days in the 4 different disease categories by treatment group during stabilized rice bran addition. No significant differences were determined for treatment group in each health category

Disease status	Group^1^		
	CTR	SRB	Total
Healthy, n (%)	569 (23.4)	629 (25.9)	1198 (49.3)
Slight, n (%)	481 (19.8)	429 (17.7)	910 (37.5)
Moderate, n (%)	130 (5.4)	168 (6.9)	298 (12.3)
Severe, n (%)	8 (0.3)	14 (0.6)	22 (0.9)
Total, n (%)	1188 (48.9)	1240 (51.1)	2428 (100)

^1^CTR Control group not exposed to heat stabilized rice bran in the diet, *SRB* Group receiving a daily dose of 120 g of stabilized rice bran corresponding to 10% of the daily calories

**Table 2:** Logistic regression results for the effect of treatment on the disease severity status^1^

Cumulative days categorized by disease severity	Odds Ratio	95% CI	*P-value*
Healthy vs. Sick	1.13	0.82–1.57	0.43
Severe vs. healthy, slight, moderate	0.65	0.16–2.58	0.54
Slight vs. moderate and severe	0.77	0.52–1.13	0.19
Severe vs. slight and moderate	0.58	0.15–2.22	0.43

^1^Control calves are considered as the reference category, compared with stabilized rice bran supplemented heifers

**Fig. 2:**
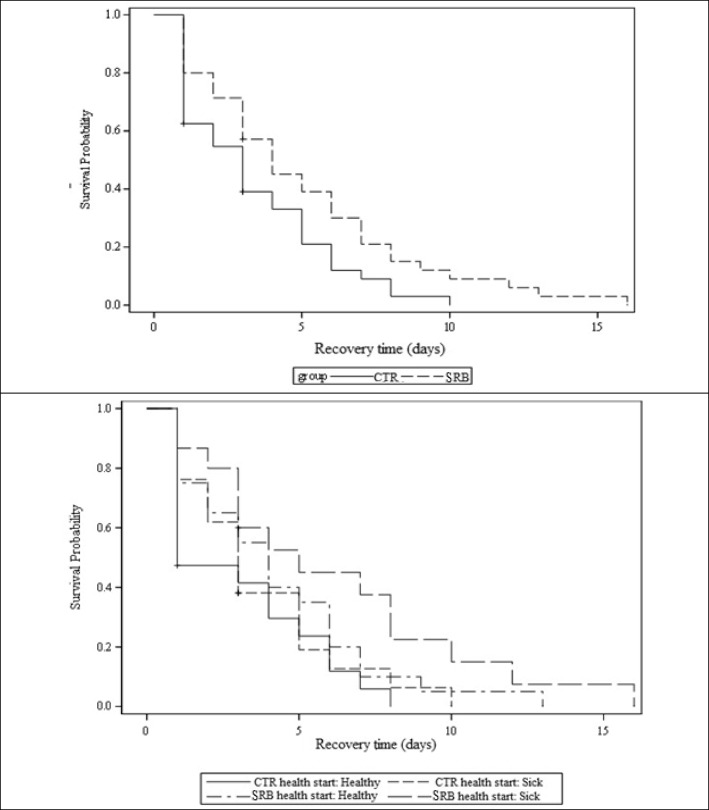
Time to recovery from a “moderate” diarrhea. **a** Treatment group (**b)** Health at enrollment and treatment group. Control (CTR; solid line) vs. Stabilized Rice Bran (SRB; dashed line) groups (*P* = 0.052). Comparison including health status at enrollment (healthy or sick) and treatment groups. Healthy CTR (solid line), healthy SRB (Dashed and dotted line), sick CTR (dashed line), sick SRB (line and dashed line), (*P* = 0.12)

The time to recovery from a “moderate” disease status to a “slight” or “healthy” status, indicated a significance trend for treatment group in the Kaplan Meier analysis (*P* = 0.052). Calves in CTR group recovered from a “moderate” status in 3.1 ± 0.4 days, while SRB calves recovered in 4.9 ± 0.7 days (Fig. 2a). Importantly, when health at enrollment was added as a covariate, there were no longer differences found in time to recovery (*P* = 0.12). The survival curve considering health status at enrollment indicated that CTR calves classified as “healthy” at d0 recovered from a moderate diarrhea episode in 2.8 ± 0.6 days; d0 “sick” CTR calves recovered in 3.4 ± 0.6 days; d0 “healthy” SRB calves in 4.3 ± 0.7 days; and d0 “sick” SRB calves recovered in 5.7 ± 1 days (Fig. 2b).

### Fecal analyses and ADG

All the fecal samples collected at enrollment and at the end of the study submitted for detection of coronavirus and rotavirus (*n* = 20) were negative. Treatment groups presented a similar ADG in the 28 days of study (CTR = 0.53 ± 0.03 kg; SRB = 0.56 ± 0.03 kg. *P* = 0.47) and the concentrations of fecal IgA did not differ between treatment groups and health status at enrollment (*P* = 0.17). Mean IgA concentrations for CTR and SRB were 3.80 ± 0.10 ng/ml and 3.54 ± 0.12 ng/ml, respectively.

### Culling, mortality and follow up period

Seventy nine calves completed the 28 days period of the study; 6 out 88 calves enrolled died (CRT = 4, SRB = 2) and 3 were culled (CTR = 2, SRB = 1). The odds of leaving the study due to death or culling did not differ between CTR and SRB group (OR = 1.93 [0.44–8.26]; *P* = 0.37). Additionally, no differences were found in the time that calves left the study due to death or culling (*P* = 0.29).

Overall, 25 (CTR = 11, SRB = 14) calves received organic certified treatment for at least one disease event during the follow up period. Ten calves (CTR = 5, SRB = 5) presented more than 1 disease event between d28 in study and weaning. No significant difference (*P* = 0.92) was found in the odds of presenting more than 2 events of disease between treatment groups (OR = 0.93, 95% CI = 0.08–1.38). In addition, the time to a first disease event after completion of SRB addition was similar in both treatment groups (*P* = 0.43).

In total, 16 out of 79 calves were lost during the post treatment follow up period, between the end of the 28 days study period and weaning. Eleven calves were sold (CTR = 5, SRB = 6) and 5 calves died (CTR = 2, SRB = 3). No differences were found in the odds of leaving the study by treatment group (*P* = 0.63, OR = 0.76, 95% CI = 0.25–2.31). Additionally, time to death or culling did not differ between groups (*P* = 0.63).

## Discussion

The addition of prebiotics via SRB into milk starting at 6–7 days of age was assessed for effects on health and performance of pre-weaned organic dairy calves over a 28 days period. Overall, this study resulted in no treatment differences in the number of days calves were sick or in the number of days by category of disease severity. Notably, the addition of SRB was tested in a challenging calf population, as the compromised health status of some calves was apparent at enrollment. The beginning of this study coincided with nutritional management adjustments made by the farm that resulted in high incidence of neonatal diarrhea.

Total serum protein determination in calves is a commonly used tool to measure passive immune transfer and, consequently, new born and colostrum management practices at farms [19]. All the enrolled calves had TSP measurements above 5.5 g/dL. It has been described that concentrations ≥5.2 g/dL in healthy calves and ≥ 5.5 g/dL in clinically ill calves is considered a measure of adequate passive transfer of immunity [18, 19]. However, the concentration of TSP in calves might be affected by dehydration and, although a cut-off point for high TSP readings in calves has not been established, readings above 7.5 have been linked to dehydration [20]. Notably, 35% of our calves presented TSP concentrations above 7.5 g/dL at enrollment, with close to 50% of the population showing signs of clinical disease (diarrhea or slight dehydration). However, no differences were found between treatment groups, indicating that both groups started in similar immune and health conditions. In addition, considering this issue, health at enrollment was included in the statistical models as a covariate.

Supporting our results, a previous study reported that the use of an oral electrolyte containing rice, promoted diarrhea in young calves less than 2 weeks old [21]. Pre-ruminant calves lack the production of enzymes to digest maltose and starch from rice and this situation might lead to osmotic diarrhea when it is provided in milk replacers or in oral electrolytes [21, 22]. This fact might explain the increase in the days SRB calves spent in the moderate and severely sick categories in our study.

Published studies using prebiotics as a prophylactic or treatment therapy in pre-weaned calves are limited and there is not consensus on their effect on health and diarrhea presentation in young dairy calves. Although positive effects were reported in the reduction of disease presentation or diarrhea scores by some authors [6, 7, 11, 13], other studies did not find significant differences [9, 10].

The decision of analyzing the time to a first “moderate” status of disease was made considering the health situation of the study population at enrollment. Calves presented a first “moderate” health condition as a result of diarrhea in the first 5 days in study, when they were 10 to 12 days old.

Treatment groups did not differ in the time to a first “moderate” status and, as it was expected, calves that were sick at enrollment showed a tendency to present the first “moderate” health status before than calves that were healthy at that time point. Contrary to our expectations, SRB calves that were healthy at enrollment presented a moderate health status earlier than healthy CTR calves. Even though these differences were not significant, we attribute this finding to a possible osmotic effect of SRB on the large intestine of young animals [21].

Our results contrast with neonatal animal model research, where SRB had a protective effect in the presentation of disease through the stimulation of the immune response and increases of probiotic bacteria in the gastrointestinal tract [14].

A tendency for different times to recovery from a “moderate” to a “slight” health status between treatment groups was established; SRB calves required more days to recover than CTR calves. This information is valuable and suggests that SRB may have a potentially detrimental effect in young calves, explained by the incapacity to digest carbohydrates and starches from rice [21].

Control calves and SRB calves had a similar ADG during the 28 days in study. Published data is not consistent on resulting ADG in calves fed with prebiotics. No differences in ADG has been reported [8, 10, 11]. Conversely, some studies found a greater ADG in pre-weaned calves fed with prebiotic (MOS) in milk for 60 days [6, 12]. Interestingly, rice protein has been used as a replacement of whey protein in milk replacers and the results are not conclusive in the effects on performance of pre-weaned calves [23, 24]. A negative impact on ADG was reported when calves were fed with rice protein replacing 50% and more of the whey protein in the milk replacer, these animals had a reduction of 12 to 54% in body weight, although health parameters were not collected [23]. Conversely, no effects on ADG or growth were reported in calves when 70% rice protein was added in the milk replacer of pre-weaned calves [24].

Immunoglobulin A is the major immunoglobulin class found in mucosal secretions and prevents mucosal infections by agglutinating pathogens [7]. Our study found similar IgA concentrations in feces from the two treatment groups. The immunomodulatory response of dietary SRB has been described in animal models. It was reported an increased production of mucosal IgA in 4 to 6 weeks old mice fed 10% of the daily calories for 28 days [15]. In that study, rice bran enhanced the growth of *Lactobacillus ssp.* and other beneficial bacteria that might have increased the IgA concentration in intestine. Similarly, an increase in the serum titer of IgA in gnotobiotic pigs fed SRB was found [14].

However, previous reports are not consistent on the immunomodulatory response of prebiotic fed to pre-weaned calves and the quantification of fecal IgA. No difference in fecal and salivary IgA was reported when newborn calves were fed for 60 days with a commercial prebiotic in milk replacer (Prebio Support, Meiji Feed Co., Ltd. Tokyo, Japan) [9]. Conversely, the same product had an effect increasing fecal IgA of pre-weaned calves at specific time points [8].

Although the CTR group had twice as many calves leaving the study as the SRB group, the odds of leaving the farm due to death or culling were not significantly different in our two groups. Contrary to the expectations, the follow up period until weaning indicated that a similar number of animals were lost in each group (CTR = 13 vs SRB = 12). Additionally, a similar proportion of calves was treated for more than 1 disease episode in the follow up period. Consequently, the 28 days of addition of SRB in the milk of calves did not have influence in health outcomes in the pre-weaned life.

Our daily dose of SRB was greater than that of published studies testing prebiotics on pre-weaned calves, where authors worked with commercial products in doses no greater than 7 g/d [6, 8–10, 12]. We offered SRB in its natural form in a dose of 120 g/d (only heat stabilized to prevent rancidity) and one difficulty observed in this trial was the necessity of an intense mixing to suspend the SRB dose in milk. Furthermore, if milk was not served soon after mixing, SRB started to decant in the bottom of the bottle, which was also reported in other study using a different product [13].

## Conclusions

The major finding from this study was that the addition of SRB in the milk of newborn calves for 28 days did not enhance performance, health, or immunity during the first month of life, a period characterized for the presentation of digestive diseases. Furthermore, no differences were found from birth to weaning in the presentation of diseases or death and removal. Further research is encouraged in older calves to investigate the potential beneficial effects of SRB at more advanced stages of life.

## Methods

The study was conducted in a commercial certified organic dairy calf rearing facility located in Northern Colorado. Calves were owned by this farm that provided consent for their inclusion in this study. Pre-weaned Holstein calves were managed during the study in accordance to the guidelines set by the Institutional Animal Care and Use Committee of Colorado State University (Protocol ID: 16-6893A).

### Animals, housing and feeding

Ninety pre-weaned Holstein heifer calves, 6 ± 1 days old, were enrolled in this research. Calves were monitored for 28 days to assess the effect of SRB addition in milk. After the 28 days feeding period, a follow up period until weaning (around 80 d of life) was completed to evaluate health outcomes based on farm records. The first stage of the study began in July 2017 and ended in August 2017. The second stage was completed in October 2017. After completion of this study, calves returned to the regular management for calves in this dairy farm.

A detailed description of the calves’ management at birth and in the rearing facility (housing, feeding, dehorning and vaccination program) was published [25]. In general, calves were immediately separated from their dam at birth, fed 2.8 L of colostrum during the first hour of life and at 3 and 8 h of life. Colostrum quality was at least 50 mg/ml IgG. After 24 h of life, calves arrived in the rearing facility and they were housed in rows of 90 individual hutches (Agri-Plastics, Stoney Creek, ON, Canada) with sand bedding and a wire panel pen attachment of 2.25 m^2^. Calves had visual but no physical contact with other animals until weaning.

Milk was provided in 2.8 L bottles (E-Z Nurse™) three times per day. During the study period the feeding schedules were 5:00 AM, 12:00 PM, and 7:30 PM. Milk collected from the hospital pen, and organic sealable milk delivered each day from an organic processing plant was pasteurized for calf feeding. Also, organic certified powder milk was provided, following preparation instructions.

Milk composition was analyzed weekly during the study period. Average ± SD fat, protein, lactose, and total solids were 3.82 ± 0.24%, 3.06 ± 0.14%, 4.56 ± 0.16%, and 12.5 ± 0.31%, respectively. Organic certified calf starter was offered to the calves from day 4 of life in clean buckets (16% Organic Calf Starter, Feedex Companies, LLC. South Hutchimsin, KS) and water was offered ad libitum since the arrival of calves.

Total serum proteins were measured by trained personal to evaluate passive transfer of immunity. A 5 ml blood sample was collected from the jugular vein in calves 3 to 7 days old in a tube without anticoagulant. The sample was allowed to clot before centrifugation. Serum was analyzed in an optical engine digital refractometer (Palm Abbe™^,^ Solon, OH) and all readings were kept in the farm recording system.

The completion of the step-down weaning process took three weeks and it was based on calf starter consumption (1.8 to 2.2 kg per day) and fully weaned calves stayed during one week in the individual hutches to monitor health before transferring to collective pens.

Trained personnel had the responsibility to perform daily health evaluations to all the calves in the facility, with the objective of detecting and monitoring sick animals to apply treatments established in the farm standard operating procedures (SOP). As the study farm is an organic certified dairy, calves that were not immediately responsive to initial treatment were sold to a conventional calf operation, where animals can receive antibiotic therapy.

### Experimental design and treatment groups

A paired comparison design with 2 treatment groups was performed. Calves were randomly assigned to a control (CTR, *n* = 45) or a treatment group (SRB, n = 45) and a clinical examination was completed to determine the health status of each calf at enrolment.

All calves were weighted at enrollment and at day 28 using a mobile platform digital scale (Caf-Cart. Raytec LLC, Ephrata, PA). This procedure was performed after the morning feeding.

A subsample of 10 calves from each group was randomly selected for fecal samples collection at enrollment and at day 28 of the study, after the morning feeding. Twenty grams of fecal matter were obtained by rectal stimulation with a gloved finger and stored in two separate sterile containers. One set of samples was submitted fresh to Colorado State University, Veterinary Diagnostic Laboratories for coronavirus and rotavirus screening. The second sample was frozen at − 20 °C for subsequent IgA analysis (IgA Bovine ELISA kit, Abnova Corporation, Taipei, Taiwan.).

A daily health assessment was performed for each calf every morning after the milk feeding. The calf health scoring chart by University of Wisconsin [26] was modified to assess fecal score. The scoring was categorized as healthy or 1 for normal feces, as abnormal or 2 for loose and pasty feces and as severe or 3 for watery feces.

Dehydration status was assessed daily using a calf dehydration chart [27]. The scores were assigned as 1 for non-dehydrated animals (< 6% water body loss) with a normal attitude, strong suckle reflex, appetite, no eyeball retraction into the orbit and skin tent lower than 2 s. Score 2 was described as moderate dehydration (6 to 8% of water body loss) were the calf was depressed with weak suckle reflex, dropped ears, dry and slightly recessed eyes into the orbit and skin tent duration of 2 to 6 s. Score 3 was described as severe dehydration (> 8% of water body loss), when the calf showed signs of depression no suckle reflex, skin tent > 6 s, dry and recessed eyes into the orbit and recumbency.

Calf attitude was assessed daily in conjunction with the health assessment. A depression scoring system to determine sickness [28, 29] was modified. Score 1 corresponded to non-depressed animals. Score 2 corresponded to calves with noticeable depression and moderate signs of weakness but without altered gait. Score 3 corresponded to calves with severe depression marked signs of weakness and altered gait, in addition calves in recumbency were included.

Approximate milk intake was recorded after the AM and PM feedings for all the calves that participated in the study. The intake was divided in 5 categories, depending on milk refusal (0%; 25%; 50%; 75%; 100%) and an average daily intake was calculated.

Each animal was assigned with a daily health severity score, based on the combined morning health assessment (diarrhea score, dehydration score, attitude score) and the average milk intake. A status of “healthy” was determined when all the scores were 1 (normal) and milk refusal was ≤25%. A “slight” disease status was applied to all the calves that had a milk refusal below 50% and at least one health score of 2. In the case of diarrhea, a score 3 was also considered “slight” when the calf was not dehydrated and its attitude was not compromised. A “moderate” disease status was applied to the calves that presented more than two health scores of 2 (or diarrhea score 2 or 3) and milk refusal above 50%. A “severe” disease status sick was given to calves in recumbency with more than two health scores in 3 and milk refusal above 75%.

### Feeding

Organic certified Jasmine Stabilized Rice Bran was provided from Urmatt Thailand as a gift from Rice Bran Technologies, Sacramento, CA (Table 3). The dose of SRB was calculated to achieve 10% of the daily total calorie intake during the first weeks of life (400 cal). This dose was calculated based on research with monogastric animals [14, 15, 17] that indicated this level of inclusion as optimal. Due to the milk feeding routine in this large rearing facility, reducing this 10% of the daily calories for the treatment group was not a possibility. The daily dose was divided into two feeding periods and mixed in the milk of the morning and the night feedings, as a higher milk intake was observed at these times compared with noon feeding. Study personnel were responsible for the mixing and feeding of treatment calves.

**Table 3:** Nutritional composition of the organic certified SRB (Rice Bran Technologies, Sacramento, CA). Guaranteed analysis provided by Cumberland Valley Analytical Services (Waynesboro, PA)

Nutrient	Concentration
Moisture	6.0
Dry Matter (DM)	94.0
Energy (kcal/100 g)	330.5
Crude Protein (% DM)	15.4
Crude Fat (% DM)	21.3
Crude Fiber (% DM)	10.0
Acid Detergent Fiber (% DM)	10.0
Neutral Detergent Fiber (% DM)	24.4
Calcium (% DM)	0.08
Phosphorus (% DM)	2.37
Magnesium (% DM)	1.10
Potassium (% DM)	1.79
Iron (PPM)	282
Manganese (PPM)	189
Zinc (PPM)	76
Copper (PPM)	4
TDN (% DM)	95.2

### Statistical analysis

Data were analyzed using SAS statistical software (9.4, SAS Institute Inc., Cary, NC USA). Calf was considered the experimental unit of analyses. Treatment group and health status at enrollment were included in the models unless otherwise specified.

Logistic regression analysis (PROC LOGISTIC) was performed to determine differences between treatment groups in the frequency of events at enrollment, during the study period, and in the follow up until weaning. Total SP measurements were categorized in two levels to detect failures in passive immune transfer or dehydration: < 7.5 g/dL and ≥ 7.5 g/dL. In addition, health status at enrollment was categorized as “healthy” or “diseased” and group differences were analyzed. These analyses were performed to assess the initial health condition of the treatment groups. Additionally, logistic regression analysis was used to determine differences in frequencies of animal removal (death and culling aggregated in one variable) between treatment groups and to analyze differences in presentation of disease (< 2 vs ≥2 or more diseases) within the follow-up period.

The association between the number of days sick (categorized by severity of disease) and treatment group was analyzed by use of repeated measures analysis for a binary response (PROC GENMOD), assuming an exchangeable correlation structure.

Total calf days “healthy” were compared with total days “sick” (combining “slight”, “moderate” and “severe”). In addition, total days calves spent with a “slight” disease condition were compared with the combination of “moderate” and “severe” days. Finally, days in “severe” condition were compared combining days with “slight” and “moderate” condition.

Time to event analysis Kaplan Meier (PROC LIFETEST) was performed to evaluate differences in time to presentation and time to recovery from the first “moderate” case of disease between the 2 groups. Additionally, time to event analysis was used to evaluate differences in the time animals were removed and to evaluate differences in time to first disease after the end of the addition of SRB. The Wilcoxon test was used to determine statistical significance.

Least square means (PROC GLM) were calculated for ADG and IgA concentration. IgA results were firstly Log10 normalized. Statistical significance was defined at *P* < 0.05. Tendency was defined at 0.05 < *P* < 0.1.

## Abbreviations

ADG: Average daily gain
CI: Confidence interval
CTR: Control group
IgA: Immunoglobulin A
MOS: Mannanoligosaccharides
OR: Odds ratio
SRB: Heat stabilized rice bran
TSP: Total serum protein
